# MXene functionalized collagen biomaterials for cardiac tissue engineering driving iPSC-derived cardiomyocyte maturation

**DOI:** 10.1038/s41699-023-00409-w

**Published:** 2023-06-27

**Authors:** Giuseppe A. Asaro, Matteo Solazzo, Meenakshi Suku, Dahnan Spurling, Katelyn Genoud, Javier Gutierrez Gonzalez, Fergal J. O’ Brien, Valeria Nicolosi, Michael G. Monaghan

**Affiliations:** 1 Department of Mechanical, Manufacturing and Biomedical Engineering, Trinity College Dublin, Dublin, 2 Ireland; 2 Advanced Materials and BioEngineering Research (AMBER), Centre at Trinity College Dublin and the Royal College of Surgeons in Ireland, Dublin, 2 Ireland; 3 Trinity Centre for Biomedical Engineering, Trinity College Dublin, Dublin, 2 Ireland; 4 CÚRAM, Centre for Research in Medical Devices, National University of Ireland, H91 W2TY Galway, Ireland; 5 School of Chemistry, Trinity College Dublin, Dublin, 2 Ireland; 6 Tissue Engineering Research Group, Department of Anatomy & Regenerative Medicine, Royal College of Surgeons in Ireland, Dublin, 2 Ireland

**Keywords:** Biomaterials - cells, Tissues

## Abstract

Electroconductive biomaterials are gaining significant consideration for regeneration in tissues where electrical functionality is of crucial importance, such as myocardium, neural, musculoskeletal, and bone tissue. In this work, conductive biohybrid platforms were engineered by blending collagen type I and 2D MXene (Ti_3_C_2_T_x_) and afterwards covalently crosslinking; to harness the biofunctionality of the protein component and the increased stiffness and enhanced electrical conductivity (matching and even surpassing native tissues) that two-dimensional titanium carbide provides. These MXene platforms were highly biocompatible and resulted in increased proliferation and cell spreading when seeded with fibroblasts. Conversely, they limited bacterial attachment (Staphylococcus aureus) and proliferation. When neonatal rat cardiomyocytes (nrCMs) were cultured on the substrates increased spreading and viability up to day 7 were studied when compared to control collagen substrates. Human induced pluripotent stem cell-derived cardiomyocytes (iPSC-CMs) were seeded and stimulated using electric-field generation in a custom-made bioreactor. The combination of an electroconductive substrate with an external electrical field enhanced cell growth, and significantly increased cx43 expression. This in vitro study convincingly demonstrates the potential of this engineered conductive biohybrid platform for cardiac tissue regeneration.

## Introduction

Scaffolds for regenerative medicine must address certain criteria in order to adequately drive cell behaviour towards achieving mature and functional tissue development and organisation^1,2^. Among some of the fundamental properties that a biomaterial for tissue engineering should have—biocompatibility and biodegradability, suitable mechanical properties, and biomimetic architecture to enhance vascularization and nutrient supply^3^—electroconductivity is a feature that is gathering significant attention in recent years for tissues where conduction of electrical stimuli is of crucial importance^1,4^.

Electroconductive materials are a relatively new player in the repertoire of biomaterials and are of interest to mimic the electrical features of native tissues such as muscle, neural, and bone^4,5^ since it has been demonstrated that they promote the proliferation and differentiation behaviour of electrical stimuli-responsive cells, such as neurons and cardiomyocytes (CMs)^4–9^. For example, the use of conductive substrates in cardiac applications; aside from stimulating cardiomyocyte growth and alignment^7,10^, can also improve the propagation of electrical impulses to synchronize beating^8^. Such an approach could obviate arrhythmias that typically occur with the non-conductive scar tissue that forms following myocardial infarction^8^. Another useful application of electroconductive materials is nerve generation where studies have demonstrated electrically conductive substrates improve neurite outgrowth, cell alignment, and nerve growth factor (NGF) gene expression of Schwann cells^7^. Bone also exhibits and responds to piezoelectricity, and electrical stimuli improve osteoblast activity with increased cytosolic calcium flow through voltage-gated channels resulting in an increment in intracellular calcium, leading to enhanced bone regeneration^6,7^.

Three main classes of conductive biomaterials—conductive polymers, carbon nanomaterials and metal nanomaterials—are being investigated and their application in tissue engineering has drawn much attention^6,11^.

These conductive biomaterials are quite easy to work with, can be manufactured in large-scale processes^6,11^, and are often incorporated together with therapeutic natural polymers. Native polymers, such as collagen, alginate and gelatin, present biological cues to enhance biomimeticity and cell interactions—and have been doped with conductive materials^4^. Taking for instance collagen; collagens are the most abundant protein in the body accounting for approximately 30% of the body’s extracellular matrix (ECM) with collagen types I, II and II being responsible for 80–90% of all collagens^12^. It is widely used in the field of tissue engineering and regenerative medicine as a natural biomaterial as it can be widely sourced and processed to maintain bioinstructive and biomimetic cues, and has demonstrated several successes in the field. Therefore it emerges as a leading choice of biomaterial in which electroconductive biomaterials could serve as a filler. Nanocomposite materials (denoted as ‘extrinsically conductive’), exist as a polymer matrix acting as an insulator, while the filler conducts electricity. When a critical amount of electroconductive particles (that reaches the electrical percolation threshold) is added to polymeric matrices, the electrically conductive particles are physically close enough to each other, forming electron-conductive paths inside the polymer composites and permitting flow of electrons and electrical conductivity of the polymer by many degrees of magnitude^13^. The material’s conductivity is low below the percolation threshold, and dramatically increases over that value^11,14^.

Carbon nanotubes^15^, graphene^16^, metallic nanoparticles^17^ and MXene^18,19^ have been shown to possess adequate biocompatibility, becoming promising materials for biomedical applications. Considering the recent advent of such materials; their approval to the clinic is being met with caution. A primary concern raised by several researchers over the safety of nanomaterials has primarily focused on their nanoforms, as nanosheet liquid suspensions or aerosols; and a key concern with the safety of implanted materials containing nanomaterials is with their release into the body in the form of nanofragments coming from implanted bulk materials, or the delamination of nanomaterials from coated surfaces^20^.

MXenes are a large family of 2D transitional metal carbides and nitrides which are derived from the layered ceramics known as MAX phases (where ‘M’ is an early transition metal, ‘A’ is usually Al or Si, and ‘X’ is C and/or N). They are a relatively new contender in this field and may have advantages over existing materials. For one; they can be mixed as an additive to elastomeric substrates with ease, and reports to date suggest excellent biocompatibility^14,21,22^, anti-microbial properties and as phototherapeutic agents in cancer treatment due to their photothermal priorities^23^. In comparison to more established 2D nanomaterials (e.g. Graphene), MXenes possess increased hydrophilicity^24^ and superior electrical conductivity^25^. The most studied MXene is Ti_3_C_2_T_x_^14,21^, which is etched from its parent MAX phase Ti_3_AlC_2_^26^. During the etching process, MXenes acquire surface functional groups, such as -O, OH, or F^27,28^ granting high hydrophilicity^21,29,30^. Furthermore, Ti_3_C_2_T_x_ is metallic, with a high density of states (DOS) at the fermi level^31^.

In this work we seize the clear benefits of MXene by engineering and optimizing an electroconductive platform for tissue engineering applications, by combining of collagen type I with MXene, bringing together the biocompatibility and biomimetic properties of the native extracellular matrix polymer^32^ with the high conductivity and enhanced mechanical properties of the MXene. With significant potential as a next-generation biomaterial substrate, this platform also supports fibroblasts culture and proliferation as well as cardiomyocytes attachment and growth, and finally iPSC-derived cardiomyocyte maturation when combined with an external electric field.

## Results and discussion

### Fabrication and morphological analysis of the biohybrid platforms

**Fig. 1:**
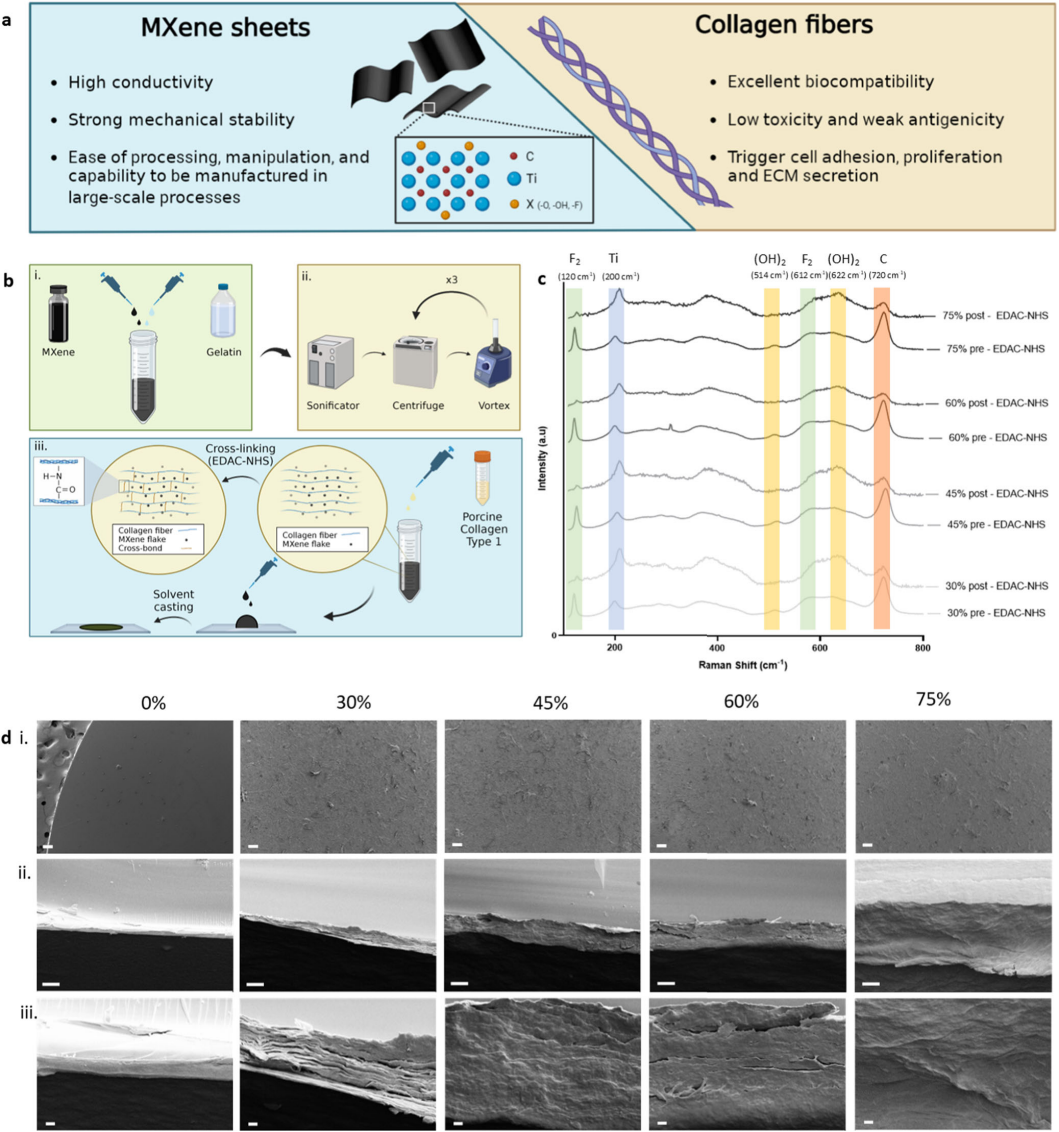
Fabrication and morphological-chemical analysis of the biohybrid platforms. **a** MXene and collagen illustration, highlighting the advantageous features of each compound; **b** schematic illustration of the blending process protocol to create the biohybrid platforms of MXene and Collagen; **c** raman spectra of the samples, highlighting the MXene element (carbon, titanium) and the surface functional groups (F, (OH)_2_); **d** SEM micrographs: i. surface view; ii–iii. cross section view (scale bar i.: 100 µm; ii.: 10 µm; iii.: 1 µm).

Ti_3_C_2_T_x_ etched using HF (Hydrofluoric Acid) exhibits an accordion-like morphology with residual forces keeping MXene layers together, therefore sonification was employed to etch into individual layers using gelatin as a surfacting agent. Scanning electron microscopy (SEM), X-ray diffraction (XRD), energy dispersive X-Ray (EDX) and Fourier transform infrared spectroscopy (FTIR) were performed on MXene flakes and films casted using gelatin as the surfactant to confirm the residual presence of gelatin on MXene after the washing steps. (Supplementary Figure 1). The use of gelatin reduces the interfacial surface tension, and facilitates generation of a homogeneous solution with collagen type I (Supplementary Figure 3). An acidic environment was exploited to enhance the electrostatic interaction between the anionic MXene flakes^33–35^ and the cationic charge that collagen fibres acquire at low pH^36–38^. After casting into 2D films (showed in Supplementary Figure 2), the stability of the films was enhanced (Supplementary Figure 4) using EDAC-NHS cross-linking to link the collagen chains closer (Fig. 1b)^39,40^. To understand the impact of MXene on this biohybrid platform, 4 groups of increasing weight percentages of MXene were studied (30%, 45%, 60% and 75% w/w MXene). For qualitative analysis of chemical groups and bonding information in the MXene-collagen structures, Raman spectroscopy was performed before and after the cross-linking process. The Raman spectra verified that in each MXene group, the typical chemical groups of MXene were present: Ti (200 cm^−1^) and C (720 cm^−1^); and the presence of functional groups: F_2_ (120 and 612 cm^−1^) and (OH)_2_ (514 and 622 cm^−1^) (Fig. 1c)^21,29,41^, suggesting that cross-linking does not affect the chemical structure of titanium carbide. MXene not only increases surface roughness of the otherwise smooth collagen film, but also contributes to a thicker 2D platforms, with an increase from a few microns for the collagen to tens of microns for higher MXene content (Fig. 1d).

### ECM stabilization of MXene platforms is concentration dependent and impact mechanical behaviour

**Fig. 2:**
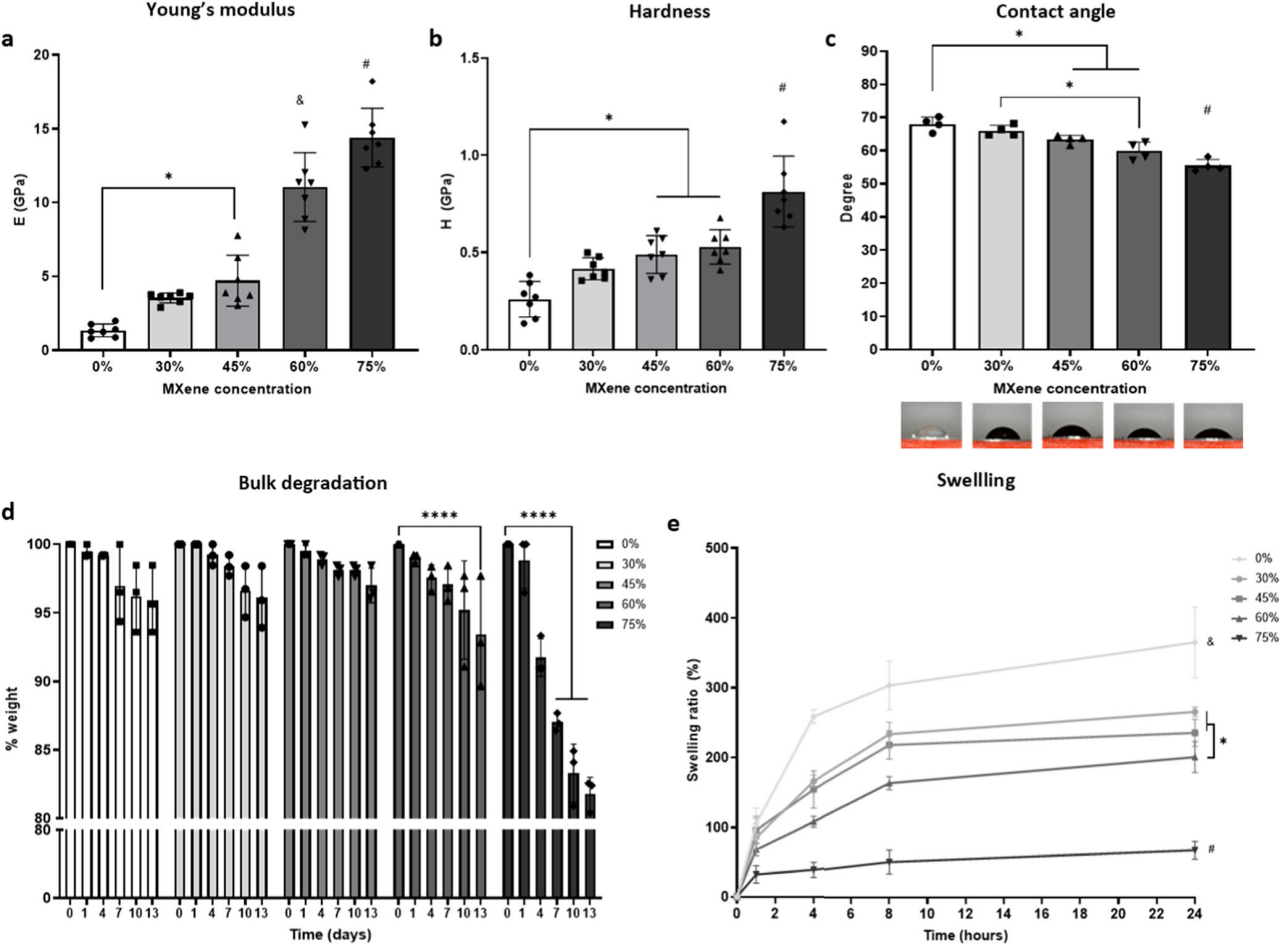
Mechanical and physical analysis. **a** Young’s modulus (GPa) of 0%, 30%, 45%, 60% and 75% w/w MXene films; **b** Hardness (GPa) of 0%, 30%, 45%, 60% and 75% w/w MXene films; **c** Contact angle (°) for 0%, 30%, 45%, 60% and 75% w/w MXene; **d** Weight percentage (%) after dissolution test, evaluated in 5 time points (1 day, 4 days, 7 days, 10 days, and 13 days) of 0%, 30%, 45%, 60% and 75% w/w MXene films; **e** Swelling ratio (%) evaluated in 4 time points (1 h, 4 h, 8 h, 24 h) of 0%, 30%, 45%, 60% and 75% w/w MXene films. Statistical analysis was performed using a one-way ANOVA with Tukey’s multiple comparison test (except for (**d**), where two-way ANOVA was performed), where a resultant *p*-value of less than or equal to 0.05 marked with * was considered significant. # and & represent statistical significance (*p* < 0.05) between the indicated group and all other groups using one-way ANOVA with Tukey’s posthoc test. All graphical data represents the mean with error bars defining ±standard deviation.

With the fabrication of the biohybrid substrates optimized, we next sought to assess the impact of MXene on mechanical and physical properties. Young’s modulus and hardness increased proportionally with the % w/w content of MXene, with an enhanced elasticity and stiffness of the biohybrid films (Fig. 2a, b) which overcomes the poor mechanical performance that is typical of biological-derived polymers in their naïve state^42^. Wettability of the films is an important criterion of biomaterials as it impacts surface proteins adsorption^43^, and consequently, cell adhesion^44,45^. Cells adhere preferentially to moderately hydrophilic surfaces, as hydrophilicity enhances protein absorption^46^. With an increasing content of MXene, substrate hydrophilicity increases (Fig. 2c), in line with previous works that attributed the hydrophilicity of MXene to surface terminal groups as hydroxyl, oxygen or fluorine^35,47^, We demonstrate that these groups are present in these biohybrid films by Raman spectra (Fig. 1b). Among other parameters that influence material interaction with cells, swellability is another key feature to be considered^48^. With increasing amounts of MXene, a statistically significant decrease in water uptake ratio was observed. (Fig. 2e). Additionally, the stability of the biohybrid films were assessed by subjecting them to a bulk degradation test where the films were incubated at 37 °C in PBS. It was found that all the groups retained more than 95% of their mass after 13 days (Fig. 2d). However, 75% w/w MXene biohybrid films exhibited a continual drop in mass which became notable from day 4, indicating that the bulk collagen network was insufficient to contain the MXene flakes within the film.

### MXene concentration is proportional to the conductivity of the biohybrid platforms in both hydrated and crosslinked states

**Fig. 3:**
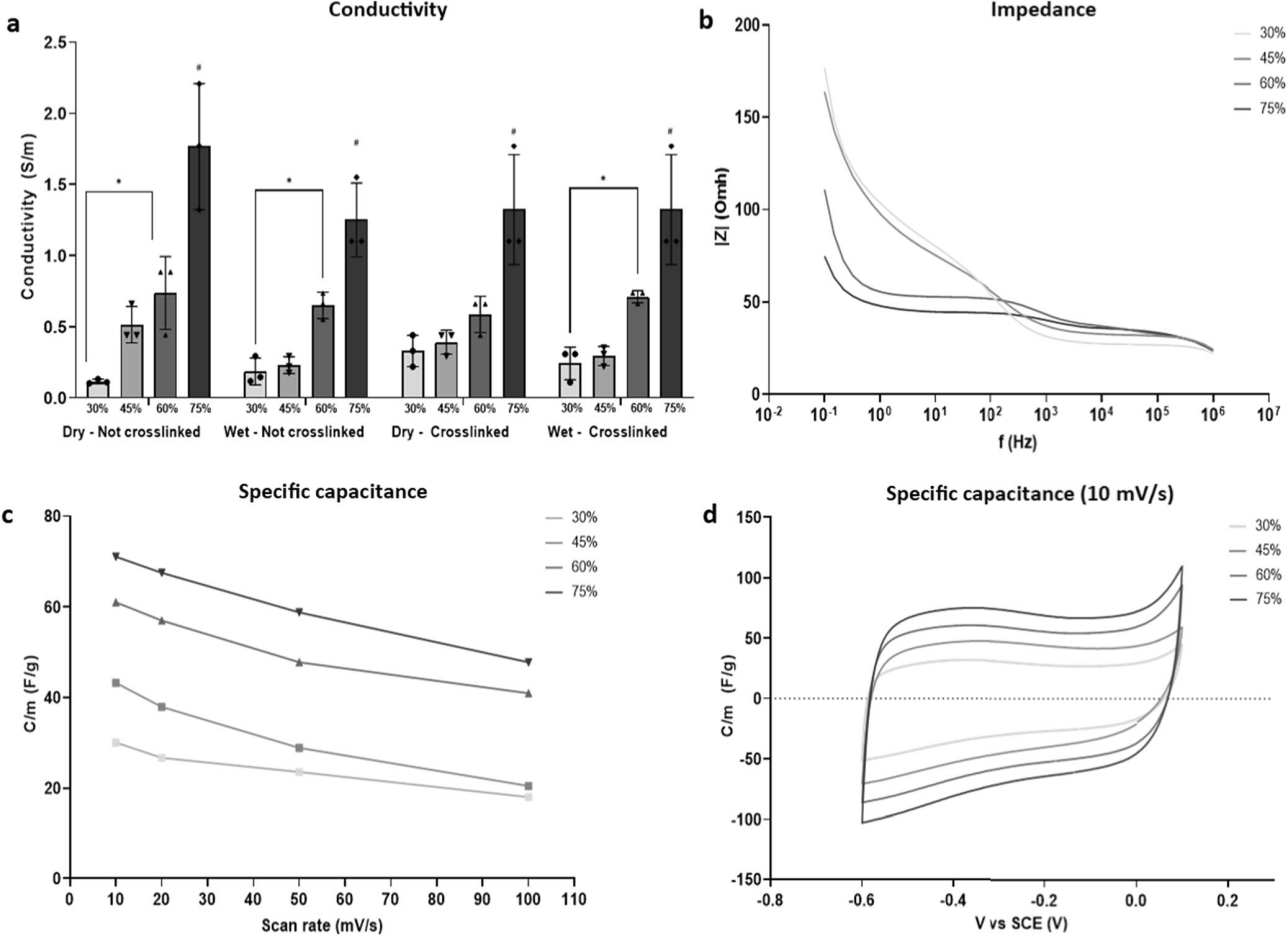
Electroconductive property. **a** Conductivity (S m^−1^) of 30%, 45%, 60% and 75% w/w MXene films, performed before and after the cross-linking step, in both cases in dry and hydrated conditions; **b** impedance (Omh) of 30%, 45%, 60% and 75% w/w MXene films; **c** specific capacitance (F g^−1^) of 30%, 45%, 60% and 75% w/w MXene films; **d** Specific capacitance curves (F g^−1^) of 30%, 45%, 60% and 75% w/w MXene films evaluated at scan rate equal to 10 mV s^−1^. Statistical analysis was performed using a one-way ANOVA with Tukey’s multiple comparison test, where a resultant *p*-value of less than or equal to 0.05 marked with * was considered significant. # Represents statistical significance (*p* < 0.05) between the indicated group and all other groups using one-way ANOVA with Tukey’s posthoc test. All graphical data represents the mean with error bars defining ±standard deviation.

With the impact of MXene on the mechanical and physical performances of the biohybrid platforms established, conductivity was assessed within four different conditions. While investigating the dependence of the electrical conductivity on the MXene content, we also assessed whether the cross-linking process could interfere with the electrical conduction, and if the hydration state (i.e. dry or wet) of the biohybrid films played a major role. The impact of MXene on conductivity is clear, and was also proportional to the MXene concentration employed, ranging from 0.1 S m^−1^ for 30% w/w MXene to over 1.5 S m^−1^ for 75% w/w MXene (Fig. 3a). No significant differences were detected between the four different environmental condition, suggesting that EDAC-NHS cross-linking of the collagen or the hydration state had a minor role in the conduction of the electrical signal. Incidentally, the conductivity of the biohybrid MXene-collagen platforms matches closely and mildly surpasses the conductivity values of native tissues such as myocardium (0.2 ~ 0.8 S m^−1^)^49–51^, skeletal muscle (0.2 ~ 0.4 S m^−1^)^52,53^ and grey matter tissue (0.1 ~ 0.3 S m^−1^)^54,55^. Upon examination, the specific capacitance at four different scan rates, noted that with increasing MXene concentrations, the capacitance also increased (Fig. 3d), highlighting the influence of MXene concentration on energy storage in this platform. Previous works have document that redox capacitance is a major contributor to MXene capacitance, causing the electrochemical adsorption of ions in the surface and consequently charge-transfer^56^. Here, it is observed that scan rate is inversely proportional to specific capacitance: as the scan rate increases, specific capacitance decreases (Fig. 3c). To understand this, slowing down the scan rate, causes electrolytes to penetrate more thoroughly into the surface area (which is large and rough/textured), making greater contact with the internal surface of the material. As a result, more charge is stored on the surface and the measured capacitance increases, becoming closer to the intrinsic capacitance of the material^57^. Similarly, the impedance of the biohybrid platforms verifies the capacitor-like behaviour of the MXene, with impedance decreasing as frequency increases. Specifically, MXene lowers the impedance of the biohybrid system, resulting in a lower opposition to current flow, and ultimately a higher conductivity (Fig. 3b). Taken together, the conductivity, capacitance and impedance characterisation clearly demonstrate the electroconductive nature of the material, confirming the strong electroconductive ability of the biohybrid platforms.

### Biohybrid platforms show high biocompatibility, allowing cell spreading and proliferation

**Fig. 4:**
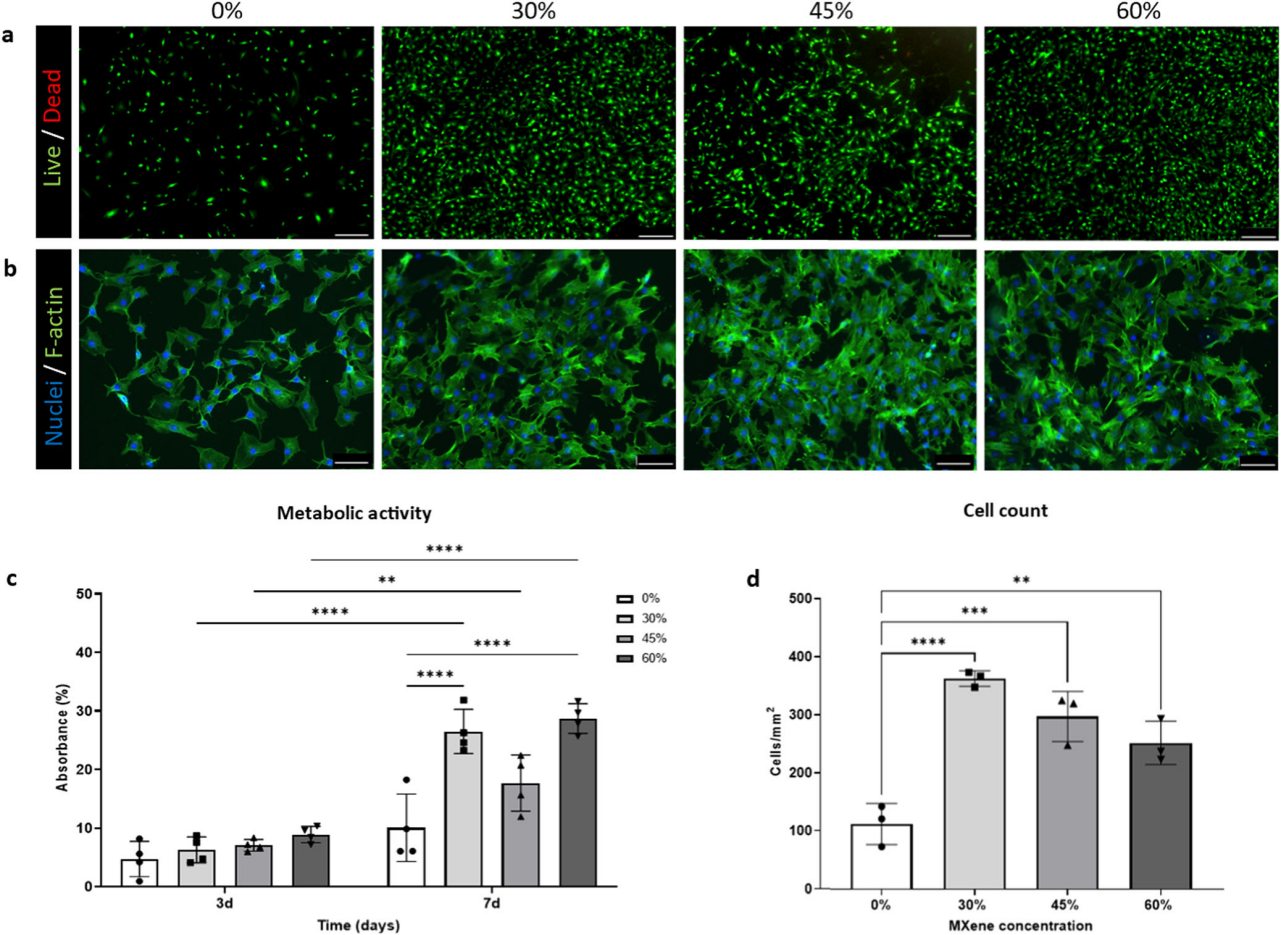
Cytocompatibility of C3H10 cells on biohybrid platform. **a** Live/Dead (scale bar: 200 µm) at day 7; **b** nuclei/F-actin staining (respectively, DAPI and phalloidin) (scale bar: 100 µm) at day 7; **c** metabolic activity which was assessed via AlamarBlue™ reduction assay at day 3 and day 7; **d** Quantification of viability (cells mm^−2^) at day 7. Statistical analysis was performed using a one-way ANOVA with Tukey’s multiple comparison test, where a resultant *p*-value of less than or equal to 0.05 was considered significant.* (<0.05), ** (<0.01), *** (0.005) and ****(<0.001) signify a statistically significant difference between the groups indicated. All graphical data represents the mean with error bars defining ±standard deviation.

To assess the initial biocompatibility of the substrates, mouse embryonic fibroblasts C3H10 were employed. Biohybrid films with 75% w/w MXene were excluded for the experiments from this point due to their instability in aqueous media at 37 °C (Fig. 2d). C3H10 cells were highly viable with the biohybrid platforms with a negligible number of dead cells at day 7 (Fig. 4a). DAPI/F-actin (for nuclei/cytoskeletal skeleton respectively) staining determined that C3H10 cell morphology was not impacted by the concentration of MXene. Nuclei retained a circular appearance, while cells spread flat on the substrates, yielding confluent monolayers with excellent cell-cell interactions (Fig. 4b). Additionally, C3H10 cells cultured in all Mxene biohybrid platforms showed a significant increase in metabolic activity (assayed using AlamarBlue™ reduction) from day 3 to day 7 (Fig. 4c), another parameter that indicates cell viability. Meanwhile, besides there being no statistically significant increase in the metabolic activity of C3H10 cells cultured in collagen from day 3 to day 7, C3H10 cells grown in MXene biohybrid platforms at day 7 show greater viability and higher metabolic activity than ones cultured in the collagen. This outcome has been confirmed by the C3H10 cell count, where the addition of MXene led to increased number of C3H10 cells on the substrates when compared with collagen (0% MXene) (Fig. 4d).

### MXene lends anti-bacterial and anti-fouling property to biohybrid platforms

**Fig. 5:**
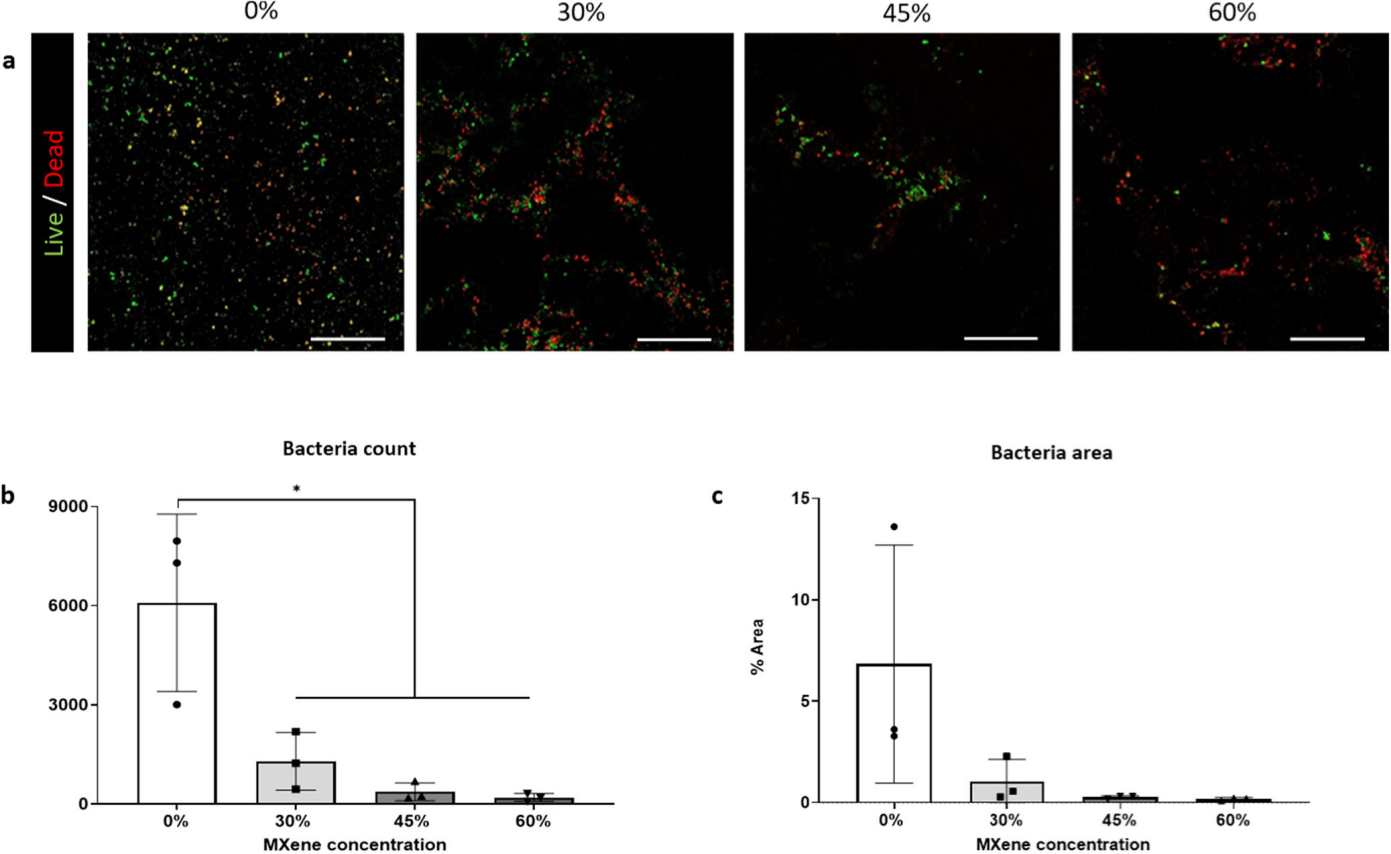
Antimicrobial evaluation of MXene/collagen type I biohybrid films. **a** Live/Dead™ BacLight™ (scale bar: 200 µm) after 24 h with live bacteria in green and red bacteria in red; **b** count of *S.aureus* Newman strain (both live and dead) attached on the biohybrid platforms; **c** field of view area of % with *S.aureus* present after 24 h. Statistical analysis was performed using a one-way ANOVA with Tukey’s multiple comparison test, where a resultant *p*-value of less than or equal to 0.05 was considered significant. * Signifies a statistically significant difference between the groups indicated. All graphical data represents the mean with error bars defining ±standard deviation.

Antimicrobial capacity of a biomaterial is a feature of importance, since bacterial infection can lead to implant failure and the development of an anti-fouling biomaterial is significant considering the advent of multidrug bacterial resistance^58,59^. The primary causative pathogen of implant related infections is *Staphylococcus aureus* and here, we found that the biohybrid films displayed inhibited gram positive *S.aureus Newman* attachment and proliferation after 24 h (Fig. 5a). With increasing MXene concentrations, a significantly lower number of bacteria attached to the biohybrid films when compared to collagen films (Fig. 5b). Similarly, the absolute area occupied by bacteria reduced with increasing MXene concentration (Fig. 5c). This outcome is in line with other previous studies^60,61^ that attribute the anti-bacterial effect to the anionic surface^[46]^ or to hydrogen interactions between the oxygenate groups of Ti_3_C_2_T_x_ MXene and the bacteria membrane’s lipopolysaccharides^47,62^.

### Biohybrid films facilitate cardiomyocyte growth

**Fig. 6:**
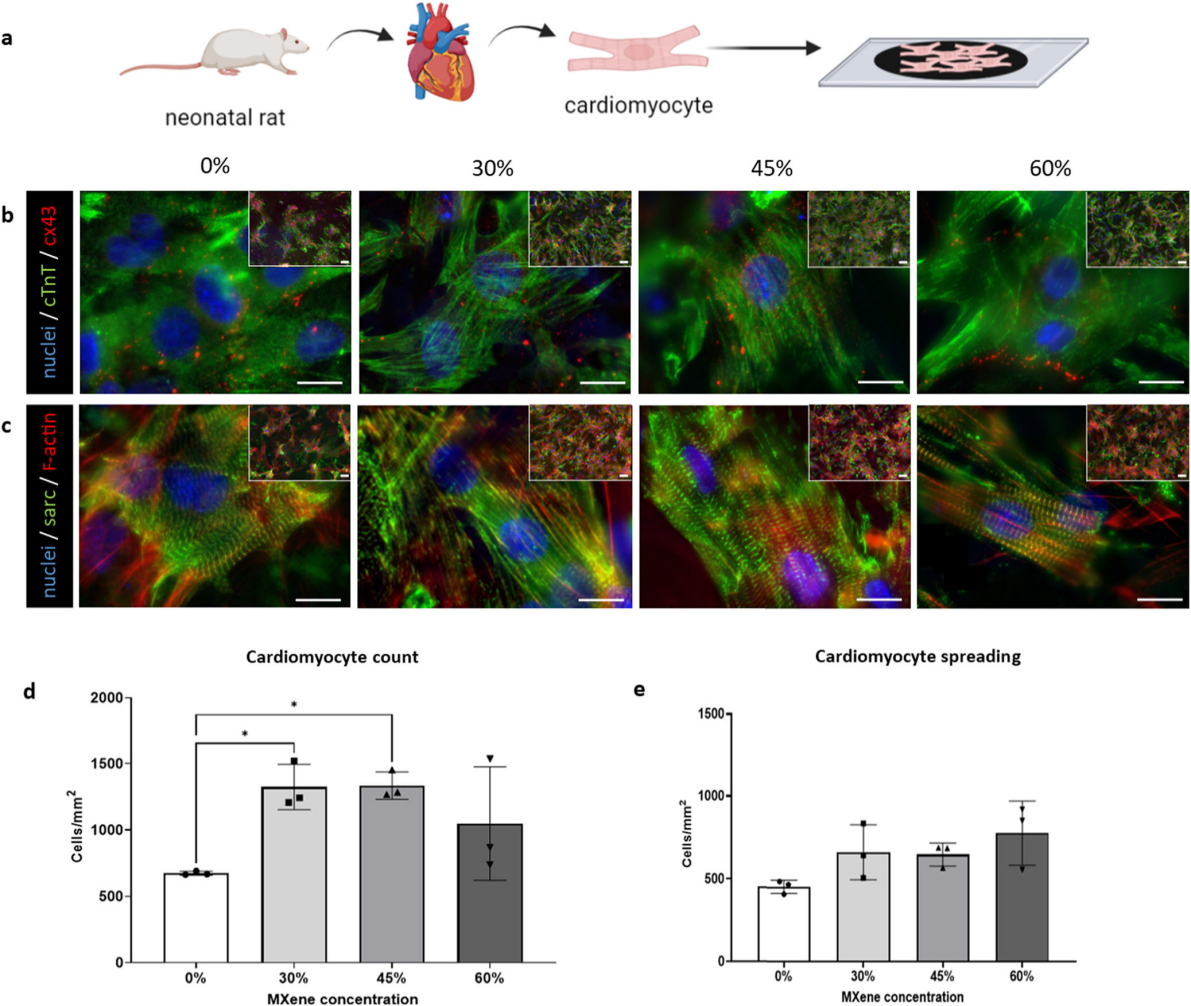
Neonatal rat cardiomyocytes study. **a** Schematic illustrating the setup of the experiment with extracted neonatal rat cardiomyocytes seeded onto biohybrid substrates containing 0%, 30%, 45% and 60% MXene and cultured for 7 days; **b** row of immunofluorescent staining micrographs with nuclei in blue (via DAPI), cardiac troponin T (cTnT) in green and connexin43 (cx43) staining in red (scale bar: 20 µm) at day 7; **c** row of immunofluorescent staining micrographs with nuclei in blue (via DAPI /Sarcomeric α-actinin green and F-actin staining by staining with Rhodamine Phalloidin (scale bar: 10 µm)) at day 7; **d** quantification of viability (cells mm^−2^) at day 7; **e** cardiomyocytes spreading quantification (µm^2^ cell^−1^). Statistical analysis was performed using a one-way ANOVA with Tukey’s multiple comparison test, where a resultant *p*-value of less than or equal to 0.05 was considered significant. *Signifies a statistically significant different between the groups indicated. All graphical data represents the mean with error bars defining ±standard deviation.

After having demonstrated the impact of MXene concentration on the mechanical, electrical and cell biocompatibility properties, as well as their antimicrobial capability, we next sought to assess the suitability of these biohybrid films for cardiomyocytes. Cardiomyocytes are responsive to electrical stimuli, and have been reported to proliferate and have improved differentiation when in contact with electroconductive biomaterials^7,10^. Therefore we extracted neonatal rat cardiomyocytes and seeded onto biohybrid substrates containing 0%, 30%, 45% and 60% MXene and cultured for 7 days (Fig. 6a). In this study, primary neonatal rat cardiomyocytes retained an expression of connexin-43 (Cx43), cardiac troponin T and sarcomeric α-actinin throughout all the groups (Fig. 6b, c). Additionally, on day 7, a significantly higher number of cells were found to be attached to the biohybrid films when compared with collagen films (Fig. 6d), with increased cell spreading correlating with an increased MXene concentrations (Fig. 6e).

### Electrostimulation through a bioreactor induces iPSC-derived cardiomyocytes to a more mature phenotype, enhancing cell elongation

**Fig. 7:**
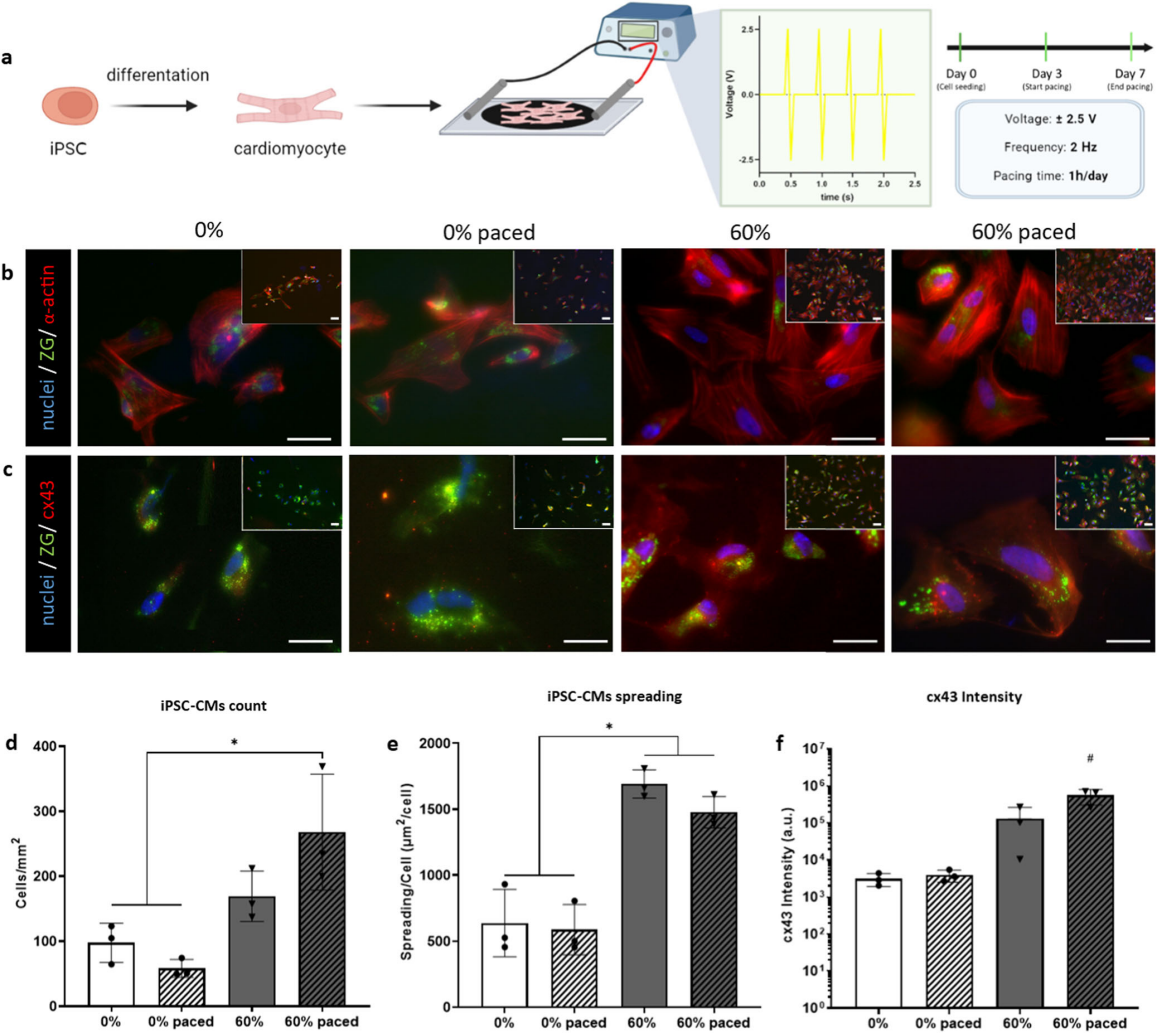
iPSC-CMs bioreactor study. **a** Schematic illustrating the experimental setup with cardiomyocytes differentiated from human induced pluripotent stem cells (iPSCs) seeded upon control (0%) and 60% MXene biohybrid films and subjected to a square wave electrical pacing regime of ±2.5 V at 2 Hz for 1 h a day over 7 days; **b** row of immunofluorescent staining micrographs of iPSC-derived cardiomyocytes under control and experimental conditions (±pacing, ±MXene) with Nuclei staining blue via DAPI, sarcomeric α-actinin in red and transgenic ZS-Green which is endogenously expressed in this iPSC-cell line (scale bar: 10 µm) at day 7; **c** row of immunofluorescent staining micrographs of iPSC-derived cardiomyocytes under control and experimental conditions (±pacing, ±MXene) with Nuclei staining blue via DAPI,Connexin43 (cx43) in red and transgenic ZS-Green which is endogenously expressed in this iPSC-cell line (scale bar: 10 µm) at day 7; **d** quantification of viability (by number of cells mm^−2^) at day 7. **e** Cardiomyocytes spreading quantification (µm^2^ cell^−1^); **f** Grey Value Intensity quantification of cx43 expression in the experimental groups (a.u.). Statistical analysis was performed using a one-way ANOVA with Tukey’s multiple comparison test, where a resultant *p*-value of less than or equal to 0.05 was considered significant. *Highlights a significant difference between the groups indicated, and # signifies a significant increase in Cx43 expression when compared to all other groups in the data set. All graphical data represents the mean with error bars defining ±standard deviation.

Having shown the significant influence an electroconductive substrate has on electrically responsive cells, we next evaluated the added impact of an external electric field, through a custom-made bioreactor^63^. Induced pluripotent stem cells-derived cardiomyocytes (iPSC-CMs) were seeded upon the substrates and an electric field for 1 h per day was applied at day 3 and each day afterwards up to day 7, using a biphasic 2 ms long pulse of ±2.5 V and a frequency of 2 Hz (Fig. 7a). iPSC-CMs preferably adhered to the MXene-embedded platforms (Fig. 7d), with increased spreading and elongation on the MXene biohybrid platforms, when compared to the collagen substrates (Fig. 7b, e). The combination of an electroconductive material with the external electric field led to an enhanced expression of cx43, reflective of improved functionality and maturation of iPSC-CMs (Fig. 7f). According to these findings, the electrically stimulated iPSC-CMs cultured on 60% w/w MXene biohybrids switch to a more mature adult cardiomyocyte phenotype when compared to the control collagen (0% MXene) substrates.

These findings are in agreement with several others, a recent example being the culture of iPSC-CMs on aerosol printed MXene on polyethylene glycol (PEG) hydrogels which led to improved conduction velocity and increased expression of maturation markers^64^. Similarly; others have explored the combined impact of MXene and electric field stimulation; most recently in one application focussed on neural stimulation and differentiation. Part of this work concluded that the combination of both MXene substrates and electric field stimulation leads to increased neural stem cell proliferation^65^. In one other related approach; Mao et al. harnessed electric field stimulation to promote wound healing in a full thickness skin defect model in a rat, whereby a cellulose/MXene hydrogel yielded increased wound healing when applied with electric field stimulation^66^. The findings of our study with MXene embedded collagen substrates together with electric field stimulation are new potent examples where this combination has a clear impact on stem cell maturation with significant potential in the field of tissue engineering and regenerative medicine.

In summary, this study demonstrates the strong potential of an engineered conductive biohybrid platform of collagen type I with MXene (Ti_3_C_2_T_x_), that exhibits high electric features, improved mechanical properties, hydrophilicity and swelling, highly biocompatible while also inhibiting bacterial attachment (S. aureus)—crucial features that next-generation medical devices must satisfy—and most significantly, facilitates and enhances attachment, elongation and maturation of iPSC-derived cardiomyocytes when an external electrical stimulation is applied. These findings are promising in the development of highly biocompatible and conductive biohybrid platforms that may be applied to a variety of biological applications requiring electrical conductivity. Furthermore, this work has demonstrated the excellent features of MXene and its prospective usage in the future as a component with natural polymers. Indeed it could be implanted into electrically conductive tissues to serve as an electrically conductive bridge or scaffold, as well as an in vitro platform to grow electrically sensitive cells outside the body.

## Experimental methods

### MXene synthesis and formulation

To a vented 200 ml PTFE vessel, deionised water (25 ml) was added, followed by drop-wise addition of 12 M hydrochloric acid (75 ml, Sigma). LiF powder (4.8 g, Sigma) was added, and the vessel was then placed in a mineral oil bath stirring at 400 rpm using a magnetic PTFE stirrer bar for 10 min to fully dissolve the LiF and allow the temperature to stabilise. Ti_3_AlC_2_ MAX phase powder (3 g, Carbon-Ukraine Ltd.) was then added in small additions to the vessel over a period of 30 min to avoid overheating of the solution. The bath was then set to 35 °C for 24 h after which was obtained etched, multilayer Ti_3_C_2_T_x_ MXene. To wash, the contents of the vessel were transferred evenly into 8 ×50 ml centrifuge tubes and diluted to a total of 40 ml each with deionised water. The dispersions were then sedimented via centrifugation at 5000 rpm using a Thermo Scientific Heraeus Multifuge X1 for 5 min, discarding the supernatant and repeating several times, until the pH of the supernatant had reached at least 6. To delaminate the washed multilayer MXene, the dispersions were transferred to a 500 ml flask and stirred vigorously for 2 h. The delaminated dispersion was then centrifuged at 1500 rpm for 30 min to sediment any many-layer MXene or unreacted MAX phase that remained. The supernatant containing delaminated MXene flakes was then collected. This supernatant was then centrifuged at 5000 rpm for 1 h to sediment the large monolayer flakes, the sediments were then redispersed in a total of 20 ml deionised water to obtain a concentrated MXene solution of approximately 40 mg ml^−1^. To determine the concentration of the MXene dispersion, 100 μl was transferred to a glass vial and diluted with a 1:1 (v/v) ratio of deionised water and absolute ethanol (Fisher) before being filtered using a pre-weighed 0.25 μm pore nitrocellulose filter membrane (Millipore VSWP) and vacuum filtration flask. The vial and sides of the funnel were washed down with additional 1:1 deionised water and absolute ethanol. Once the filtration was complete the membrane and MXene filtride were dried overnight in a vacuum desiccator and then weighed to obtain the concentration.

### Scanning Electron Microscopy (SEM)

Biohybrid platforms were mounted on aluminium stubs with a conductive carbon tape (Ted Pella, USA), and a gold–palladium layer of approximately 5 nm was sputter coated on the sample surface. Characterisation with SEM was performed using a Zeiss Ultra Plus (Carl Zeiss) microscope fitted with a Gemini column, SE detector and ESB detector. The typical acceleration voltage used was 1.5 kV. Energy dispersive x-ray spectroscopy (EDX) spectra were acquired using the EDAX detector at an acceleration voltage of 15 kV, and acquired in the range of 0–10 kV.

### X-ray diffraction

Films were characterized with XRD, using a Bruker D8 Discovery X-ray Diffractometer in θ/2θ configuration, in the range of 3 − 75°, at 2° min^−1^. D-spacing was calculated from the Ti3C2Tx (002) reflection according to the formula for Bragg’s law:

$$n\lambda =2{d\,sin}\theta$$

### Fabrication of MXene-collagen biohybrid platforms

Since Ti_3_C_2_T_x_ MXene presents forces binding that keep flakes together, to reduce the surface tension a gelatin solution (Sigma-Aldrich) was used as surfactant. Gelatin facilitates the formation of a colloidal solution when collagen and MXene are mixed. Gelatin was dissolved at 40 °C to obtain a solution 4 mg ml^−1^, and sterilized by autoclaving at 120 °C. The MXene stock solution etched using HF, at concentration of 40 mg ml^−1^ was added to the gelatin solution to obtain a final concentration of 20 mg ml^−1^. The solution was homogenised by vortex and sonification, then 3 repeated washings were carried out by centrifuging the mixture at 5000 rpm for 45 mins at room temperature to centrifuge down MXene flakes, decant the excess of gelation in the supernatant and resuspended to wash again.

Porcine collagen type I was dissolved in 0.05 M acetic acid (HAc) (pH ≈ 3) overnight at a *T* < 6 °C under rotation at 20 rpm. The collagen solution, with a concentration 15 mg ml^−1^, was neutralized using approximately 1 μl 10 M sodium hydroxide (NaOH). Then, the MXene/Gelatin solution at 20 mg ml^−1^ concentration was mixed with the collagen solution. MXene-Collagen solutions were made at 30%, 45%, 60% and 75% w/w MXene, maintaining for each group a final solution concentration of 15 mg ml^−1^. Finally, the solution was vortexed before being drop casted to ensure equal dispersion of the MXene. 200 μl of solutions were drop casted in the glass coverslips with a diameter of 12 mm, then they were annealed in the oven at 40 °C for 2 h.

### Collagen crosslinking

To stabilize films EDAC (1-Ethyl-3-(3-dimethylaminopropyl)carbodiimide) and NHS (N-Hydroxysuccinimide), was used to covalently crosslink the collagen structure as described in previously works with minor changes^67^.

### Bulk degradation

Dry samples were submerged in 1x Phosphate Buffered Saline (1xPBS) at 37 °C with the media refreshed each day. Following incubation, samples were washed twice with distilled water and dried in the oven. The percentage change in weight at each time point was evaluated according to the following equation (Eq. 1):

1 $$\% {\rm{Swelling}}=\frac{{{\rm{W}}}_{{\rm{s}}}-{{\rm{W}}}_{{\rm{t}}}}{{{\rm{W}}}_{{\rm{t}}}}\times 100$$

where W_0_ is the dry starting mass and W_t_ is the dry mass at time t. The percentage of weight loss was evaluated at 5 different time points (1 day, 4 days, 7 days, 10 days, and 13 days).

### Swelling test

Dry samples were submerged in X1PBS and incubated at 37 °C and refreshed every day. Following incubation, samples were washed twice with distilled water. The swelling ratio of each sample was calculated using the equation (Eq. 2):

2 $$\% {\rm{Swelling}}=\frac{{{\rm{W}}}_{{\rm{s}}}-{{\rm{W}}}_{{\rm{d}}}}{{{\rm{W}}}_{{\rm{d}}}}\times 100$$

where W_s_ and W_d_ are the swollen weight and the dry weight respectively. The swelling ratios were evaluated at 3 different time points (1 h, 4 h, 8 h, 24 h).

### Contact angle measurement

Water contact angle was measured through a custom-made 3D printed instrument that allows to place a droplet of 10 μl of DI-water on a glass slide coated with biomaterial films. The droplet was allowed to settle for 10 seconds before taking a photograph using a Dino-Lite digital microscope (Dino-lite AM4113ZT USB Microscope). Images were analysed in ImageJ to determine contact angle.

### Conductivity measurement

The electroconductivity measurement was carried out through four point method as previously discussed^68^ using a custom built setup available open source^69^.

### Electrochemistry characterization

Samples were prepared by drop casting the MXene/Collagen dispersion onto indium tin oxide (ITO) coated glass. Electrochemical measurements consisting of cyclic voltammetry (CV) and electrochemical impedance spectroscopy (EIS) were carried out using a VMP-300 potentiostat (Bio-Logic, France). CVs were obtained at scan rates from 10 to 100 mV s^−1^, with potentiostatic EIC measurements carried out at 0 V and −0.5 V, using a frequency range of 1 MHz to 100 mHz and sinus amplitude of 10 mV.

### Raman spectroscopy

Raman spectra were acquired in backscattering configuration using an inVia confocal Renishaw Raman microscope equipped with diode (785 nm) lasers excitation. The incident beam was focused by a Leica microscope with a 50x magnification objective and short focus working distance; incident power was kept <2 mW to avoid sample damage. Spectra were baseline corrected using commercial software prior to analysis. The mapping was performed by raster scanning in the streamline mode at various *x*-axis steps.

### Nanoindentation

Nanoindentation was performed using Nano Indenter XP (Now, Keysight Technologies), a mechanical microprobe system that automatically evaluates the hardness and the elastic modulus. an increasing load from 0 to 10 mN, with an increasing step of 0.5 µN was applied.

### Cytocompatibility

To assess the biocompatibility of the material, direct cytotoxic tests were performed. C3H10 mouse embryonic fibroblasts were cultured in growth media prepared using Dulbecco’s Modified Eagle’s Medium (DMEM) low glucose (Sigma-Aldrich) containing 10% v/v fetal bovine serum (FBS) (Gibco® by Life Technologies) and 2% v/v penicillin streptomycin (Pen-Strep) (Sigma-Aldrich) at 37 °C with 5% CO2. Films were sterilized with multiple washings in 70% ethanol and exposed to UV light. Following rinsing with PBS, they were incubated in the growth media for 24 h. 20,000 cells were seeded on to the samples and media was replaced once every two days, up to day 7. Cell viability, through live-dead assay, was evaluated at day 3 and 7 with the live and dead assay adopting a solution of 2 μl ml^−1^ ethidium monodimer-1 and 0.5 μl ml^−1^ calcein and 2 μl ml^−1^ in PBS. The films were observed using a scanning confocal microscope. Metabolic activity, through AlamarBlue™ assay, was carried out according to the manufacture’s protocol at day 3 and 7 by adding the AlamarBlue cell viability reagent (Invitrogen by Thermo Fisher Scientific) directly to the samples to obtain a 9:1 ratio of cell culture media to AlamarBlue™. In addition, a negative control was included that consisted solely of DMEM low glucose with 10% FBS and 1% Pen-Strep, and 10x of the reagent. The control will eliminate the effect of background absorbance from the cell culture media. The samples were incubated for 4 h at 37 °C with 5% CO_2_. Approximately 100 μl aliquots from each well were transferred into a 96-well plate and placed into a plate reader (BioTek, Synergy HT) to read the corresponding absorbance at 570 and 600 nm.

To visualize the morphology of cells in the films, staining was carried out to detect nuclei and F-actin filaments. C3H10 cells were cultured on samples with MXene concentrations of 0%, 30%, 45% and 60%. At day 7 the cells were fixed in 4% paraformaldehyde (PFA) for 10 min. Films were permeabilized with 0.5% Triton X-100, which was dissolved in PBS, by incubation at room temperature for 5 min. Following incubation, films were washed three times in PBS, with a 5-min incubation between each wash. The working solution was prepared by combining 2 μl of phalloidin (1 μl ml^−1^), 2 μl of 4’,6-Diamidine-2’-phenylindole dihydrochloride (DAPI) (1 mg ml^−1^) (Sigma-Aldrich) and 2 ml of PBS together. Both the nucleus and F-actin filaments can be shown under fluorescent imaging. The working solution was added on top of the films to completely submerge the scaffold in the solution. Samples were protected from light and incubated for 30 min. They were then washed three times in PBS before being mounted on glass slides. Glass slides were covered with microscope cover glasses and edges were sealed with clear nail polish. Fluorescent imaging was carried out with a fluorescent microscope.

### Anti-bacterial study

The anti-bacterial properties of the films were assessed using S. aureus Newman (gram positive), a clinical isolate of osteomyelitis, at a concentration of 5 × 10^5^ CFU ml^−1^ as recommended by the Clinical and Laboratory Standards Institute. The films were seeded with 5 × 10^5^ CFU ml^−1^ of S. aureus Newman in Brain Heart Infusion (BHI) broth and incubated at 37 °C for 24 h prior to beginning LIVE/DEAD and alamarBlue™ assays. The antibiofilm and contact-kill potential of the MXene films against S. aureus Newman bacteria was evaluated using LIVE/DEAD™ BacLight™ bacterial viability kit for microscopy & quantitative assays (Invitrogen, USA) was used as per the manufacturer’s protocols following 24 h of S. aureus culture on the films in BHI broth. After culture, the films were rinsed three times with PBS, fixed using 10% formalin solution for 30 min at 4 °C, followed by three more rinses with PBS. The films were incubated with the stain for 3 min at room temperature and then rinsed again with PBS. The stained scaffolds were imaged using confocal microscopy and analysed using ImageJ to determine the percentage of bacterial cell area on the scaffolds.

### Ethics approval

Ethics approval for extraction of neonatal rat cardiomyocytes was granted by the Trinity College Dublin Animal Research Ethics Committee as part of a governing approval for extraction of primary cells from neonates. Ethical approval for the use of commercial iPSCs is not necessary in Trinity College Dublin.

### Isolation and handling of neonatal rat cardiomyocytes

Primary CMs were isolated from neonatal rat hearts (nrCM) using a protocol adapted from the Pierce Primary Cardiomyocyte Isolation Kit from Thermo Scientific (88281, Thermo Fisher Scientific). After isolation, cells were seeded immediately on films. Cells were seeded at a density of 1 × 10^6^ cells per film and were cultured in the Pierce Primary Cardiomyocyte culture media.

Immunofluorescent staining for cardiac markers was performed with an internal protocol at day 7. Briefly, the cell membrane was lysed in 1 v/v% Triton X-100 (T8787, Sigma-Aldrich), then samples were blocked with a PBS-based solution consisting of 4 w/v% BSA (A2153–50G) for stabilisation, 0.05 v/v% Triton X-100 to enhance permeabilisation, 0.05 v/v% Tween20 (P1379, Sigma-Aldrich) to reduce surface tension. Cardiac troponin T (cTnT, ab8295 Abcam), connexin 43 (Cx43, ab11370 Abcam) and sarcomeric α-actinin (α-act, EA53 Thermo Fisher) were used as primary antibodies and diluted 1:300 (cTnT and Cx43) or 1:100 (α-act) in antibody dilution buffer containing PBS with 1 w/v% BSA, 0.05 v/v% Triton X-100, 0.05 v/v% Tween20. Secondary antibodies were diluted 1 in 250 in antibody dilution buffer. The secondary antibodies used for this study were Alexa Fluor® 594 for Cx-43 (ab150076, Abcam) and Alexa Fluor® 488 for cTnT and α-act (ab150113, Abcam). Rhodamine Phalloidin (00027, VWR) at concentration of 5 µ ml^−1^ was incubated together with the secondary antibody for α-act. DAPI solution was made from a 1000X stock concentrate (32670, Sigma-Aldrich). All washings were performed with PBS and 0.05 v/v% Tween20. Micrographs were obtained using an OlympusIX83 epifluorescence microscope (Olympus, Germany). Cell spreading was calculated analysing Nuclei—Sarcomeric—α-actinin staining images through ImageJ.

### iPSC culture and cardiomyocyte derivatizatio

iPSCs (SFCi55-ZsG—tagged with ZS Green^70^) were expanded in MTESR media (Stem Cell®) grown on Matrigel™ coated plates and cultured to 70–80% confluency before splitting at a ratio of 1:5 using Versene (Gibco®). Cardiomyocyte differentiation was induced using the extensively described GiWi protocol^71^, Briefly, 90–95% confluent iPSCs (day 0) were treated with 10 µM CHIR99021 in RPMI media supplemented with B27 minus insulin (differentiation media) for 24 h. On day three, the cells were treated with 5 µM IWP2 for 48 h, in differentiation media. On day five, the media containing IWP2 was replaced with un-supplemented differentiation media and finally from day seven, the cells were cultured in RPMI media supplemented with B27 with insulin (maintenance media). Spontaneous beating was noted from day eight of the differentiation and the iPSC-derived cardiomyocytes were lifted and seeded on the substrates on day twenty-one of the differentiation.

### Electric field stimulation

Starting from day 3, iPSC-CMs seeded on substrates were stimulated with a custom-made bioreactor^63^ with a biphasic 2 ms long pulse of ±2.5 V and a frequency of 2 Hz. Cells were stimulated 1 h per day until day 7. Immunofluorescent staining for cardiac markers (Cx43 and Sarcomeric α-actinin) was performed at day 7 (mentioned above). Cell spreading, cell count and cx43 intensity were calculated analysing, respectively, Nuclei—Sarcomeric—α-actinin and cx43 staining images through ImageJ.

### Statistical analysis

All statistical analysis was carried out using GraphPad Prism 9. Results are expressed as the mean ± standard deviation with a sample size of four (*n* = 4) unless otherwise stated. Statistical analysis was performed using accordingly, a one-way or a two-way ANOVA with Tukey’s multiple comparison test, where a resultant *p*-value or less than or equal to 0.05 was considered significant and indicated where appropriate.

### Supplementary information

Supporting Information

## Data Availability

The data that support the graphical presentations and findings within this paper are available from the corresponding author upon reasonable request.
