# Supplementary material for: MXene functionalized collagen biomaterials for cardiac tissue engineering driving iPSC-derived cardiomyocyte maturation
Source: NPJ 2D Mater Appl. 2023 Jun 27;7(1):44. doi: 10.1038/s41699-023-00409-w (PMC11041746; doi:10.1038/s41699-023-00409-w)
Supplement: Supplementary file 1 — Supporting Information [file 41699_2023_409_MOESM1_ESM.pdf]

Supporting Information

MXene functionalized collagen biomaterials for cardiac tissue engineering driving iPSC-derived cardiomyocyte maturation

*Giuseppe A. Asaro[1,2], Matteo Solazzo[1,3], Meenakshi Suku[1,3,4], Dahnan Spurling[2,5], Katelyn Genoud[2,6], Javier Gutierrez Gonzalez[2,6], Fergal J. O'Brien[2,3,6], Valeria Nicolosi[2,3,5], and Michael G. Monaghan[1,2,3,4]**

1. Department of Mechanical, Manufacturing and Biomedical Engineering, Trinity College Dublin, Dublin 2, Ireland

2. Advanced Materials and BioEngineering Research (AMBER), Centre at Trinity College Dublin and the Royal College of Surgeons in Ireland, Dublin 2, Ireland

3. Trinity Centre for Biomedical Engineering, Trinity College Dublin, Dublin 2, Ireland

4. CÚRAM, Centre for Research in Medical Devices, National University of Ireland, H91 W2TY Galway, Ireland

5. School of Chemistry, Trinity College Dublin, Dublin 2, Ireland

6. Tissue Engineering Research Group, Department of Anatomy & Regenerative Medicine, Royal College of Surgeons in Ireland, Dublin 2, Ireland.

Email: monaghmi@tcd.ie

Keywords: (tissue engineering, cardiac, electroconductive, 2D nanomaterials, scaffold, biomaterials, iPSCs)

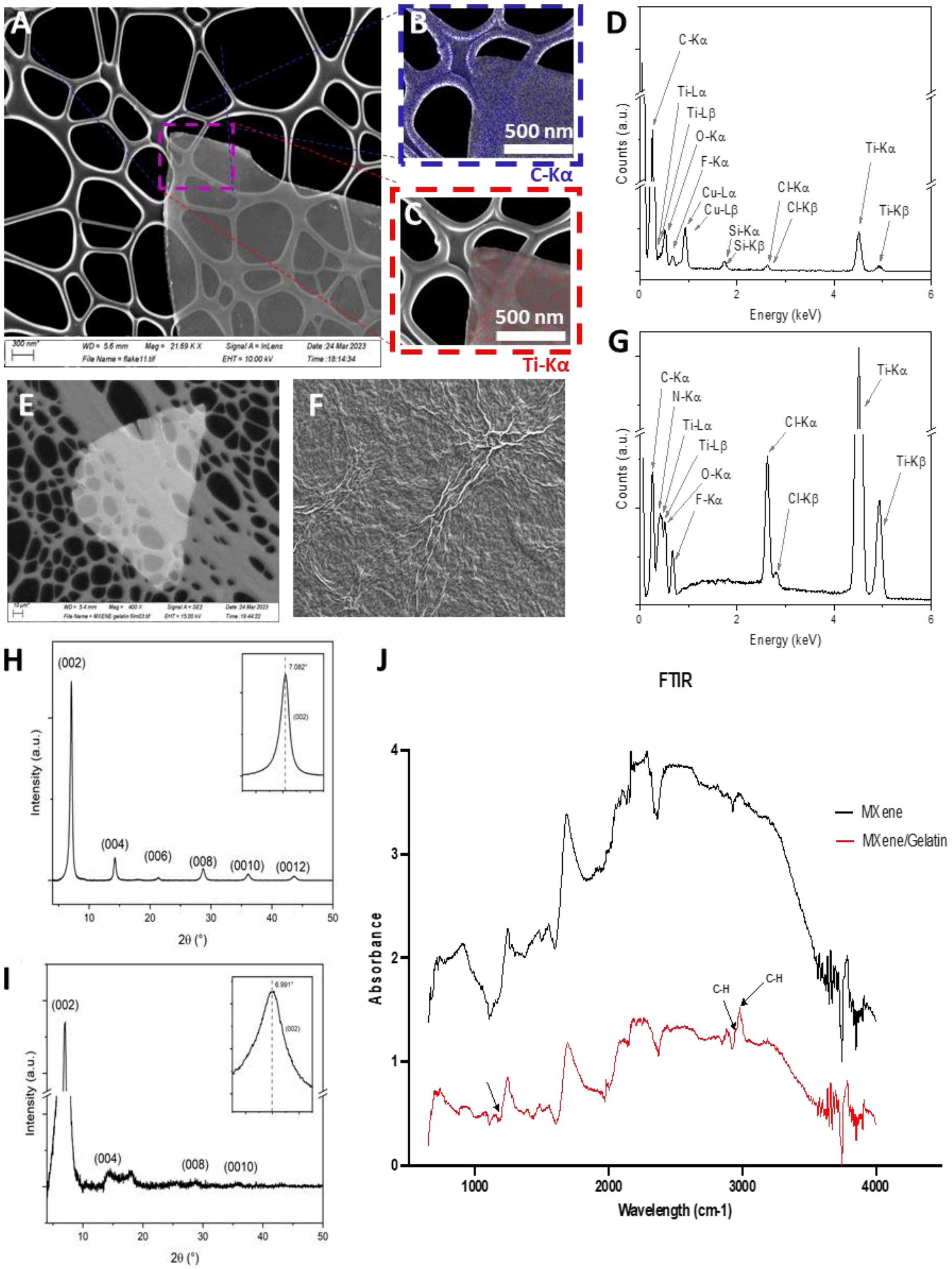

Supplementary Figure 1. (A) Energy dispersive X-ray (EDX) mapping analysis of MXene flakes highlighting Carbon-C in blue (also present in the grid) and Titanium-Ti in red. (D) sum of the EDX spectra highlighting the carbon and titanium spectra, with minute signal from Silicon-Si which emanates from the detector itself which is typical of such thin samples. (E) Gross overview of MXene flake. (F) SEM of MXene/Gelatin cast film with (G) corresponding

EDX analysis. Nitrogen-N line evident due to gelatin presence and overlap with Ti-L lines. (H) X-Ray diffraction (XRD) of MXene flakes exhibiting a typically clean (001) reflection. (002) at 7.082 gives d-spacing of 12.48 Å, interlayer spacing of 3.08 Å, based on dry multilayer spacing of 9.40 Å [S1]. (I) XRD of MXene|Gelatin cast films with a shift down in MXene (002), giving an increase in on average (12.64 Å), interlayer spacing increases to 3.24 Å, most likely due to gelatin between MXene layers. Peak at $2\theta = \sim 18$ is attributed to the presence of MXene [S2]. (J) FTIR analysis of MXene biohybrid platform demonstrating the presence of peaks characteristic to gelatin such as 2938 cm^{-1} and 2968 cm^{-1} representing C-H bonds, as well as 1160 cm^{-1} representing carboxyl-group stretching vibration.

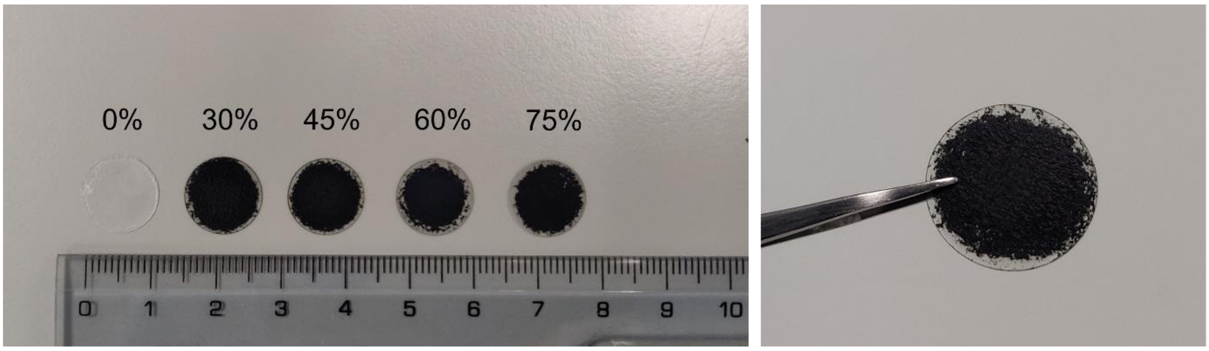

Supplementary Figure 2. Biohybrid platforms casted on glass coverslips.

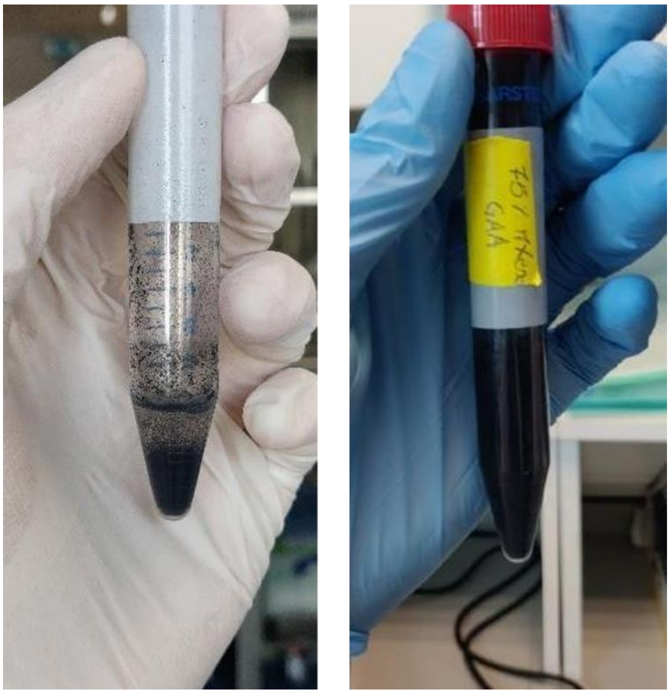

Supplementary Figure 3. On the left, an inhomogeneous solution of collagen and MXene; on the right a more uniform solution of collagen and MXene when gelatin is added as a dispersant/surfactant to MXene prior to mixing with solubilised collagen .

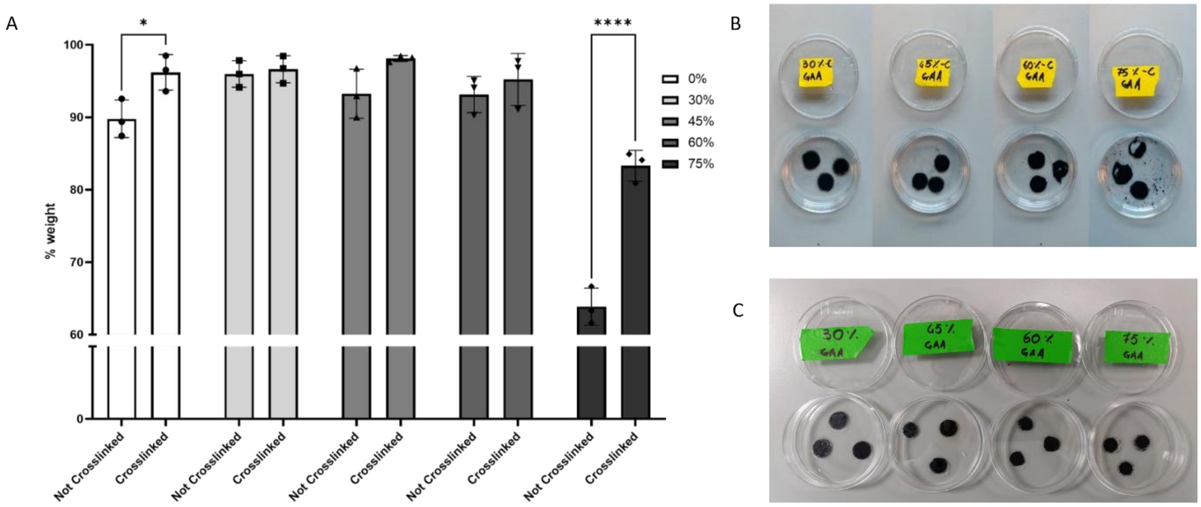

Supplementary Figure 4. Dissolution test comparison between not crosslinked biohybrid platforms and crosslinked ones. A) Weight percentage (%) after degradation test of not cross-linked and cross-linked of 0%, 30%, 45%, 60% and 75% w/w MXene biohybrid platform at day 10. B) Micrographs of non-crosslinked biohybrid platforms after 10 days. For 75% w/w MXene biohybrid platforms many floating particles can be observed in PBS. C) Micrographs of cross-linked biohybrid platforms after 10 days. Statistical analysis was performed using a one-way ANOVA with Tukey's multiple comparison where a resultant p-value or less than or equal to 0.05 was considered significant. Bars represent the mean whereby error bars depict ± standard deviation.

Supporting Information References:

1. Mikhail Shekhirev, Christopher E. Shuck, Asia Sarycheva, Yury Gogotsi, *Prog Mater Sci* **120**,100757, (2021)

2. Wang, K., Wang, W., Ye, R., Xiao, J., Liu, Y., Ding, J., Zhang, S. and Liu, A. *J. Sci. Food Agric* **97**, 3613-3622, (2017)
